# A novel presentation of *Mycobacterium avium* complex in a recipient of a lung transplant: a case report

**DOI:** 10.1186/s13256-017-1392-2

**Published:** 2017-08-28

**Authors:** Leah Cohen, Jeannette Guarner, William R. Hunt

**Affiliations:** 1 Division of Pulmonary, Allergy, Critical Care & Sleep Medicine, Emory University School of Medicine, 1365 Clifton Road NE, The Emory Clinic, Building A – Suite 3244, Atlanta, GA 30322 USA; 2 Department of Pathology and Laboratory Medicine, 1364 Clifton Road NE, Atlanta, GA 30322 USA

**Keywords:** Lung transplant, Nontuberculous mycobacterium, Endobronchial mass, *Mycobacterium avium* complex, Immunosuppression, Case report

## Abstract

**Background:**

Lung transplantation remains an important potential therapeutic option for end-stage lung disease. It can improve quality of life and in some cases be a life-lengthening therapy. Despite the possible benefits, there are also many potential complications following transplantation. Here we describe a novel presentation of nontuberculous mycobacterium manifesting as an endobronchial mass developing 4 years after lung transplantation.

**Case presentation:**

A 66-year-old African-American woman presented with progressive dyspnea, cough, and persistent wheezing of 2 months’ duration. She had a distant history of breast cancer and received bilateral lung transplantation due to end-stage pulmonary fibrosis 4 years prior to her current presentation. She denied fevers, but did endorse night sweats. She had diffuse expiratory wheezing on auscultation. Chest computed tomography imaging showed an endobronchial soft tissue lesion nearly occluding the left mainstem bronchus, which was concerning for endobronchial carcinoma. Rigid bronchoscopy demonstrated a fibrinous mass protruding into the left mainstem proximal to the anastomosis. A pathology report noted fragments of partially necrotic granulation tissue in addition to scant fragments of focally ulcerated bronchial mucosa. Both the tissue culture and bronchial wash stained positively for acid-fast bacilli and grew *Mycobacterium avium* complex.

**Conclusions:**

Nontuberculous mycobacterium pulmonary disease is common post lung transplant and risk factors are related to immunosuppression and history of structural lung disease. *Mycobacterium avium* complex presenting as an endobronchial lesion in a patient post lung transplant is a novel presentation.

## Background

Lung transplantation remains an important therapeutic option for end-stage lung disease. It can improve quality of life and, in some cases, can be a life-lengthening therapy. As such, the number of lung transplants worldwide continues to increase steadily with 3973 performed in adults in 2014 [1]. However, despite the desired benefits, lung transplantation is fraught with very serious potential complications. Early graft dysfunction, severe infections, and large airway complications such as development of exophytic granulation tissue and bronchial stenosis are common postoperatively [2].

Depending on the center, the incidence of airway complications associated with lung transplantation ranges from 7 to 18% and results in a mortality rate of 2 to 4% [3–5]. Airway complications can directly result from architectural changes associated with surgical manipulation. However, preoperative and postoperative pulmonary infections also pose a significant risk factor for airway compromise.

The increased risk for airway infections can be contributed to pharmacological immunosuppression, airway devascularization, decreased cough reflex and mucociliarly clearance, disruption of lymphatic drainage, and altered alveolar phagocytic function following transplantation [3]. Common bacterial infections include *Pseudomonas aeruginosa* and *Staphylococcus aureus*, which usually present as tracheitis, bronchitis, or pneumonia [2, 6, 7]. Endobronchial anastomotic sites are particularly susceptible to post-transplant infections, especially in the early stage following surgery [4]. *Aspergillus* can commonly cause an endobronchial infection at the anastomotic site or tracheobronchitis. Some endobronchial infections can present as a tumor-like mass, including focal cytomegalovirus (CMV) infection and *Mycobacterium tuberculosis* (TB) [5]. Infections with nontuberculous mycobacteria (NTM), such as *Mycobacterium avium* complex (MAC), are also relatively common following lung transplantation and have been associated with increased mortality [8, 9]. Manifestations of NTM following lung transplantation have ranged from asymptomatic isolation to cutaneous symptoms to severe pulmonary parenchymal disease [8–10]. However, presentation of MAC as an endobronchial lesion is very rare and has not yet been described post lung transplantation [11–13].

## Case presentation

A 66-year-old African-American woman, a recipient of a bilateral lung transplant with distant history of breast cancer, presented to our clinic with progressive shortness of breath, wheezing, and cough of 2 months’ duration. Approximately 20 years prior to undergoing lung transplantation, she developed infiltrating ductal carcinoma of her right breast with metastases to her right axilla. She underwent mastectomy and right axillary lymph node dissection and subsequent adjunct chemotherapy with cyclophosphamide, methotrexate, and 5-fluorouracil along with radiation therapy. She responded well to therapy and remained in remission. However, she also developed an aggressive form of pulmonary fibrosis and ultimately required bilateral lung transplantation secondary to end-stage lung disease 4 years prior to her current presentation.

Her subsequent medical course following transplantation had been complicated by pancytopenia requiring monthly filgrastim injections, chronic gastroparesis, hypertension, stage IV chronic kidney disease, and diabetes. Our center’s typical immunosuppression protocol utilizes a combination therapy of a calcineurin inhibitor, a cell-cycle inhibitor, and oral steroids. However, this patient’s immunosuppression regimen comprised only a calcineurin inhibitor (tacrolimus) and low-dose prednisone. She had not been on a cell-cycle inhibitor, such as azathioprine, for several years due to her significant pancytopenia.

**Fig. 1:**
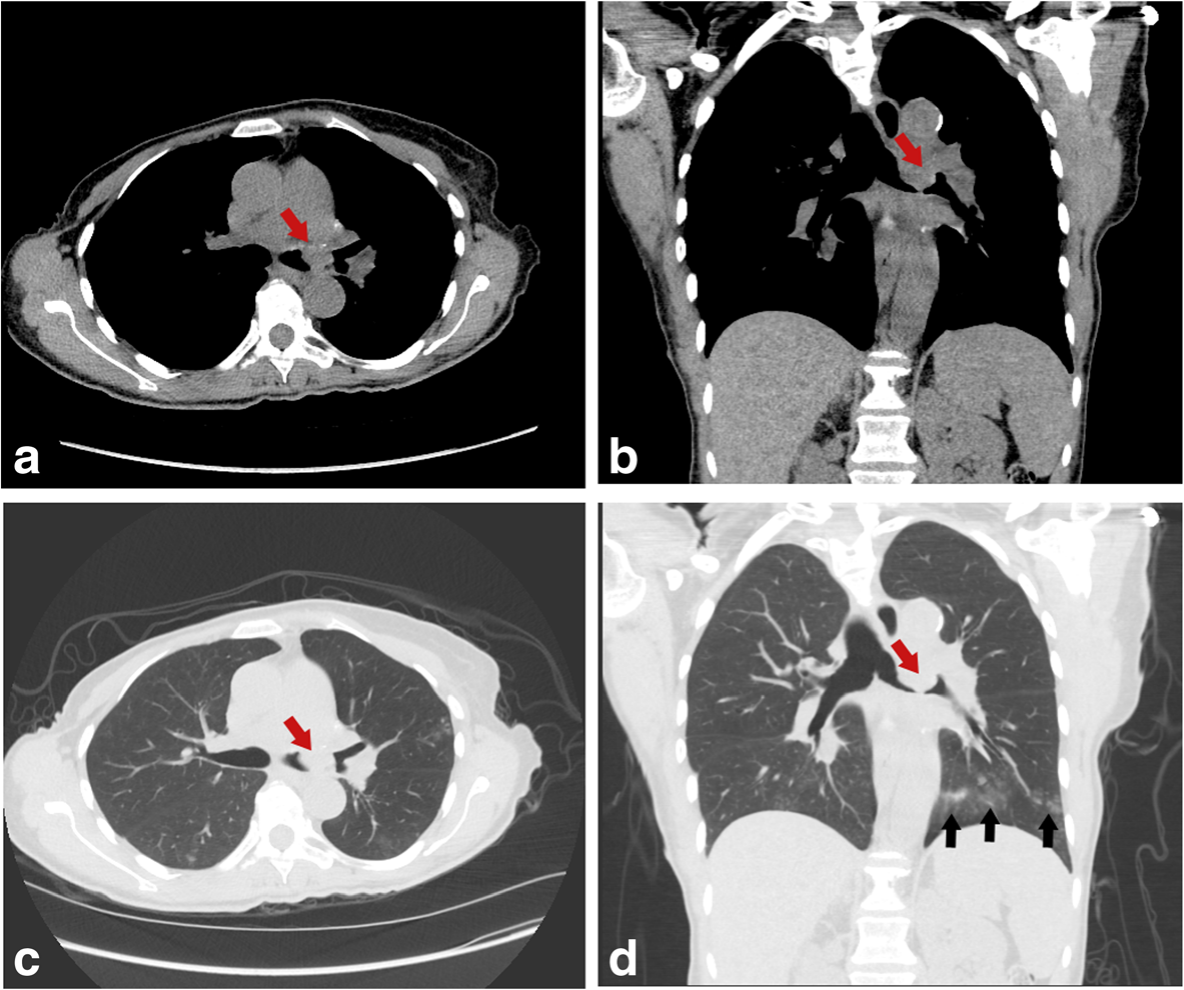
Representative computed tomography axial **a** and coronal **b** mediastinal windows which demonstrate a 2.2×1.1×0.9 cm polypoid soft tissue lesion projecting from the roof of the left mainstem bronchus (*red arrow*) adjacent to the bronchial anastomotic site. There is severe narrowing of the left mainstem bronchus with the residual lumen measuring approximately 3 mm. Computed tomography axial **c** and coronal **d** lung windows demonstrate diffusely decreased attenuation of the left lung parenchyma, compatible with diffuse air trapping secondary to obstructive endobronchial lesion. There is lower lobe predominant tree-in-bud nodularity (*black arrows*)

Upon presentation she was independently mobile, but had developed a significant decrease in her exercise tolerance. She also had developed an associated cough over the preceding 4 weeks that she reported was intermittently productive of scant blood-tinged sputum. She denied any unintentional weight loss or objective fevers, but she did endorse consistent night sweats for nearly 6 months prior to admission. She had developed persistent wheezing, only minimally responsive to bronchodilators, and chest tightness. Her nephrologist had recently discontinued her ramipril (1.25 mg daily) due to increasing serum creatinine levels. She denied any improvement in her cough with the discontinuation of the angiotensin-converting-enzyme (ACE) inhibitor. In terms of her active medications, she was on a modified immunosuppression regimen as previously described with tacrolimus 5 mg every 12 hours (goal tacrolimus trough level was between 8 and 10 ηg/mL), and prednisone 5 mg daily. The remainder of her medications consisted of aspirin 162 mg daily, atorvastatin 10 mg nightly, filgrastim 300 mcg by subcutaneous injection once weekly, pentamidine 300 mg inhaled once monthly for *Pneumocystis jirovecii* prophylaxis, and insulin therapy with Levemir (insulin detemir) 12 units subcutaneously every morning and aspart 4 units subcutaneously with meals. She was a lifelong non-tobacco smoker and had discontinued alcohol consumption prior to her lung transplantation. She held a master’s degree in education and worked as a school counselor for 30 years, but unfortunately had to take early retirement approximately a year before her lung transplantation due to her worsening lung disease. Since undergoing lung transplantation she had not traveled outside the southeastern United States of America (USA). She denied any recent sick contacts or other exposures. Her initial chest computed tomography (CT) scan is shown in Fig. 1 and demonstrated a new endobronchial lesion in the left mainstem near the anatomic anastomosis.

### Physical examination

On physical examination she was afebrile (35.9 °C) with a heart rate of 80 beats/minute, blood pressure (BP) of 137/86 mmHg, respiratory rate of 22 breaths/minute, and oxygen saturation of 97% on room air. There was no palpable cervical lymphadenopathy. A chest examination revealed a well-healed clamshell incision from her previous lung transplant surgery. There were surgical changes associated with her previous right-sided mastectomy and flap repair. There were focal end-expiratory wheezes noted over her left lung-field on auscultation. Her neuro examination was non-focal. The rest of the physical examination was unremarkable.

### Laboratory findings

Laboratory findings noted pancytopenia: white blood count of 0.9 10^3^/mL, hemoglobin 10.4 g/dL, and platelet count of 134 10^3^/mL. Serum chemistry was normal except for an elevated creatinine of 2.37 mg/dL and blood urea nitrogen (BUN) of 35 mg/dL. Blood levels of CMV and Epstein–Barr virus (EBV) were undetectable by polymerase chain reaction (PCR). Her tacrolimus level was 9.7 ƞg/dL (her goal target was 8 to 10 ƞg/dL). Her C-reactive protein was mildly elevated to 9.43 mg/L (normal range 0.3 to 8.0 mg/L).

It is protocol at our center that patients undergo serial surveillance bronchoscopies post lung transplant to evaluate for occult rejection. However, surveillance bronchoscopies are only performed routinely through the first 12 months following transplantation, with subsequent bronchoscopies performed only for clinical suspicion of underlying pathology. As such, this patient had not undergone bronchoscopy within the preceding 4 years. Her last bronchoscopy demonstrated no evidence of an endobronchial lesion, stenosis, or rejection. However, with her new symptoms and radiographic findings, there was a high concern for endobronchial squamous cell carcinoma and so she underwent rigid bronchoscopy.

**Fig. 2:**
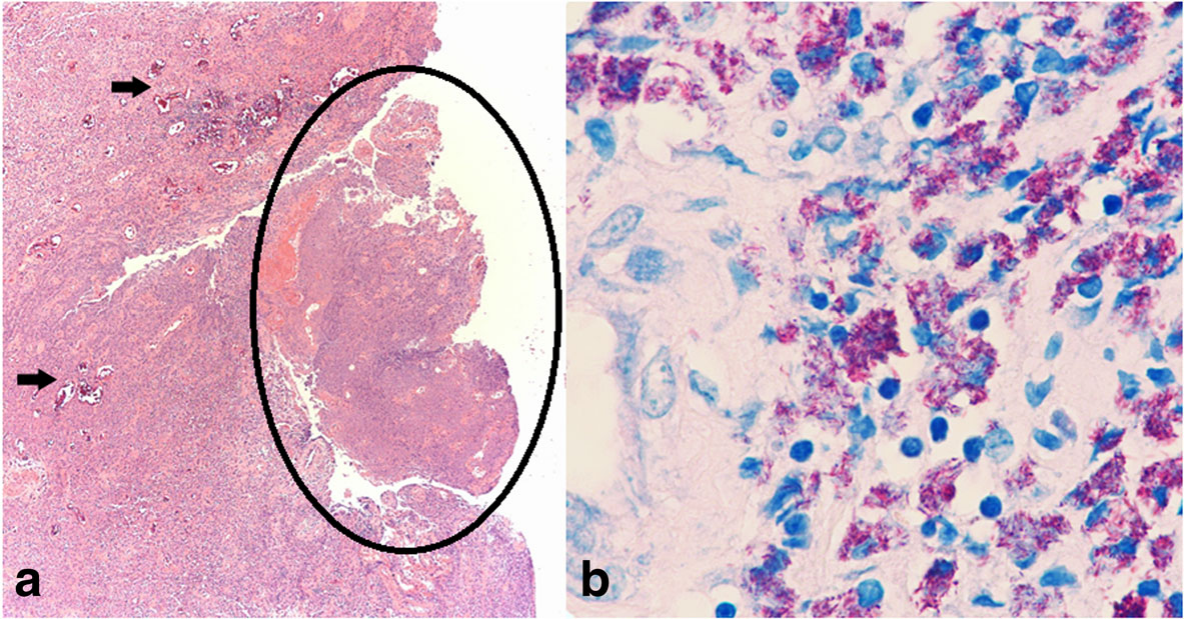
**a** Hematoxylin and eosin stain (original magnification 40×) of biopsy specimen showing granulation tissue (vessels observed to the *left*, *black arrows*) with inflammation composed of mostly macrophages with few neutrophils (*circled*). The macrophages have abundant cytoplasm with blue staining material inside them. Also present intermingled with the inflammatory infiltrate are areas of necrosis. **b** Acid-fast bacilli stain (original magnification 400×) showing abundant bacilli (all red stained material) inside macrophages

Diagnostic bronchoscopy demonstrated a fibrinous mass protruding into the left mainstem proximal to the anastomosis. Multiple biopsies were obtained from the mass and samples were sent for both histopathologic as well as microbiologic evaluation. The mass was further cored out during rigid bronchoscopy and when patency of her left-sided airway was secured, a flexible bronchoscope was inserted and a bronchial wash was performed in the lower lobe of her left lung. The bronchial wash was sent for microbiologic evaluation including bacterial, fungal, and acid-fast bacilli (AFB) culture per center policy. Histopathology demonstrated fragments of partially necrotic granulation tissue with extensive inflammation. There were also scant fragments of focally ulcerated bronchial mucosa as seen in Fig. 2. Given the pathologic findings, special stains including Gram stain, Grocott-Gomori methenamine silver stain (GMS), and AFB stain were employed on the tissue specimen. GMS staining demonstrated intracellular bacilli. The AFB stain also highlighted numerous intracellular AFB. The ill-formed granulomas and presence of little necrosis aroused suspicion that the infection was probably not tuberculosis. However, it is not possible to define the species of mycobacteria from the histopathological perspective. Therefore, a rapid Mycobacterium tuberculosis deoxyribonucleic acid (DNA) probe was performed on the bronchial wash, which was negative. Both the tissue culture and bronchial wash grew MAC.

### Therapeutic course

Given her immunosuppressed state and unusual presentation of infection, she was considered to have severe disease. She was started on therapy with azithromycin 250 mg daily, ethambutol 15 mg/kg per day, and rifampin 600 mg daily. However, out of concern for possible significant interaction with rifampin and her calcineurin inhibitor, the rifampin was exchanged for moxifloxacin 400 mg daily nearly immediately. Her immunosuppression regimen was maintained throughout her MAC therapy as she was already on a modified regimen due to her pre-existing chronic pancytopenia. As such, she remained on tacrolimus with a goal trough level of 8 to 10 ηg/mL and low-dose prednisone at 5 mg daily. She continued on triple therapy with azithromycin, ethambutol, and moxifloxacin for the first 2 months, although she tolerated the regimen very poorly. She experienced significant nausea and anorexia, losing approximately 6.8 kg (15 lbs) since just prior to her initial presentation. She also developed visual disturbances with bilateral optic neuritis approximately 2 months after initiation of therapy and ethambutol was promptly discontinued. She remained on dual therapy with azithromycin and moxifloxacin for the remainder of her antibiotic course, although she continued to have intermittent nausea, diarrhea, and poor appetite. After several negative sputum cultures, her therapy was discontinued approximately 12 months following initiation. Subsequent chest CT imaging demonstrated resolution of the nodular infiltrates in the lower lobe of her left lung and a patent left mainstem bronchus. Following discontinuation of her therapy, her visual disturbances nearly resolved as did her diarrhea. Her pulmonary function test over the 12 months following therapy discontinuation remained relatively stable and she did not develop recurrent respiratory symptoms. Unfortunately, her long-standing chronic kidney disease secondary to calcineurin inhibitor use and diabetes did progress over time. Approximately 7 months following completion of her MAC therapy she developed the need for hemodialysis. She died from complications of end-stage renal disease and hemodialysis 6 months later.

## Discussion

Complications post lung transplantation raise concern for a broad differential diagnosis depending on the timing after lung transplant. The risk for serious infections is greatest within the first year following transplantation, whereas risk and prevalence of other morbidities, such as chronic rejection and malignancy, is greater after the first year postoperatively [1]. Because our patient was 4 years out of transplant, our greatest concern was that the endobronchial mass would be some type of malignancy, such as squamous cell carcinoma or an unusual presentation of post-transplant lymphoproliferative disorder due to her chronic immunosuppression [14]. There has even been a case report of non-Hodgkin’s lymphoma presenting as an endobronchial mass in a patient with cystic fibrosis (CF) 6 years after bilateral lung transplant [15].

Of importance though, while infectious complications are more common in the first year following lung transplant, they can still occur at any time in the setting of immunosuppression. A group from Duke University published a case series in 2005 of three patients with CMV endobronchial infection presenting as a focal polyp on bronchoscopy [5]. All three patients had received lungs from CMV-positive donors, although not all patients were CMV seronegative prior to transplant. However, all three cases were discovered within the first few months after transplantation. TB has also been the cause of an endobronchial mass in the middle lobe of the right lung in a case of a 27-year-old patient with CF 3 months post lung transplant [16].

NTM presenting as an endobronchial mass is very rare. There have been a few case reports in scenarios outside lung transplantation that have described NTM presenting as an endobronchial lesion. Several case reports describe this presentation in individuals immunocompromised with human immunodeficiency virus (HIV) [11, 12]. A third case report recounts this unusual presentation in a 34-year-old Chinese woman without any evidence of immunosuppression [13]. However, while risk factors such as immunosuppression and airway architectural changes certainly increase the risk for NTM infection, presentation of NTM as an endobronchial mass following lung transplantation has not been previously described.

Although the list of NTM that cause disease in humans has increased with the larger number of immunosuppressed patients, MAC continues to be the most commonly isolated [17]. NTM are found in the soil and water, and humans acquire them from the environment, although the actual source is rarely identified. NTM most commonly presents as pleuropulmonary disease in patients with structural lung disease such as chronic obstructive pulmonary disease (COPD), CF, non-CF bronchiectasis, and post-lung transplantation [17].

NTM infection occurs in 0.46 to 9% of recipients of lung transplant with varying clinical presentation [9, 18]. Patients post lung transplant may be completely asymptomatic and have no radiologic evidence of disease with NTM cultured during routine surveillance bronchoscopy [17]. Many transplant centers, including our own, routinely perform surveillance bronchoscopies to evaluate for clinically silent acute rejection [19]. There has been ample literature describing the risk of bronchoscopic contamination with *Mycobacterium* and risk for transmission [20]. Great care must be taken to ensure pathologic contamination does not occur via diagnostic interventions, particularly in this immunocompromised population. Fortunately, in this case our patient had not required diagnostic bronchoscopy for many years prior to this presentation. NTM infection in the setting of lung transplantation can also be seen with isolated bronchiectasis secondary to bronchiolitis obliterans syndrome (BOS) or chronic rejection. The diagnosis of NTM pulmonary disease requires: a single NTM isolate from a bronchoalveolar lavage or pulmonary biopsy, or isolation of NTM from two separate sputum samples; or clinical pulmonary disease with concomitant radiographic findings [21].

There is a wide range of chest radiographic findings in NTM pulmonary disease. The cavitary or classic form appears similar to TB on chest radiograph with upper lobe cavitary lesions and endobronchial spread as evidenced by nodules adjacent to foci of disease, atelectasis, and pleural thickening [17]. Unlike TB, the cavities are usually smaller and thin walled. The bronchiectatic or nonclassic form has chest radiographic findings of randomly distributed nodular opacities. CT commonly shows small centrilobular nodules or tree-in-bud opacities with cylindrical bronchiectasis usually in the same lobe [17]. Disseminated disease is commonly seen in patients with HIV and reported radiographic findings include unifocal and unilateral alveolar opacities, mediastinal and hilar lymphadenopathy, cavitation, and pleural effusions [17]. In immunocompromised patients without HIV, such as those post lung transplant, pulmonary infections can manifest as nodules or masses within the lung tissue parenchyma [17]. MAC presenting as an endobronchial tumor-like mass has not been described as a common radiographic presentation in patients post lung transplant.

Treatment of NTM disease depends not only on the species isolated, but also on the patient’s symptoms as well as clinical presentation. In the case of MAC, the treatment of patients with nodular bronchiectatic disease usually involves a three-times weekly regimen of a macrolide, ethambutol, and rifampin [17]. For those with cavitary disease or severe nodular/bronchiectatic disease it is recommended to add streptomycin or amikacin early [21]. The treatment should continue for 1 year after a patient becomes culture negative. Sputum cultures are usually collected on a monthly basis to monitor patient response. If MAC is cultured again within the 1 year treatment window, the treatment clock restarts [21]. Because treatment involves a complicated three-drug regimen, therapy for patients who are asymptomatic may not be indicated, especially when they have adverse effects or intolerance to the drugs. However, in the case of a recipient of a lung transplant, treatment is usually recommended given the risk of associated rejection [8]. Our patient initiated three-drug therapy as described above, but due to significant side effects associated with the ethambutol as well as drug–drug interaction with rifampin and her immunosuppression medications, her regimen was adjusted to dual therapy with a macrolide and fluoroquinolone. While she had radiographic resolution of her disease as well as multiple negative sputum cultures, her duration of therapy only lasted for 12 months due to significant gastrointestinal side effects. She was subsequently monitored very closely without return of infectious symptoms. However, this case highlights the difficulties that can arise with an aggressive drug regimen and real-world practicalities compared to clinical recommendations.

## Conclusions

Airway complications following lung transplant are relatively common [3]. In addition, lung transplantation is associated with an increased risk for the development of *de novo* cancers [22, 23]. Given that our patient presented with a new endobronchial mass years after she underwent bilateral lung transplantation, we initially were very concerned for an endobronchial malignancy. However, subsequent biopsy and pathology identified MAC as the etiology of her airway mass. Risk for NTM infection is higher in immunocompromised individuals; however, in individuals following lung transplantation, NTM infection typically presents as pleuropulmonary disease. To the best of our knowledge, this is the first case of NTM presenting as an endobronchial lesion in a recipient of a lung transplant.

## Abbreviations

ACE: Angiotensin-converting-enzyme
AFB: Acid-fast bacilli
BOS: Bronchiolitis obliterans syndrome
BP: Blood pressure
BUN: Blood urea nitrogen
CF: Cystic fibrosis
CMV: Cytomegalovirus
COPD: Chronic obstructive pulmonary disease
CT: Computed tomography
EBV: Epstein–Barr virus
GMS: Grocott-Gomori methenamine silver stain
HIV: Human immunodeficiency virus
MAC: *Mycobacterium avium* complex
NTM: Nontuberculous mycobacteria
PCR: Polymerase chain reaction
TB: *Mycobacterium tuberculosis*
